# The Botanical Glycoside Oleandrin Inhibits Human T-cell Leukemia Virus Type-1 Infectivity and Env-Dependent Virological Synapse Formation

**DOI:** 10.35248/1948-5964.19.11.184

**Published:** 2019-08-21

**Authors:** Tetiana Hutchison, Laçin Yapindi, Aditi Malu, Robert A Newman, K Jagannadha Sastry, Robert Harrod

**Affiliations:** 1 Laboratory of Molecular Virology, Department of Biological Sciences, The Dedman College Center for Drug Discovery, Design & Delivery, Southern Methodist University, Dallas, Texas, 75275-0376, USA; 2 Department of Experimental Therapeutics, The University of Texas M.D. Anderson Cancer Center, Houston, Texas, 77054, USA; 3 Departments of Immunology and Veterinary Sciences, The University of Texas M.D. Anderson Cancer Center, Houston, Texas, 77054, USA

**Keywords:** HTLV-1, HIV-1, Envelope glycoprotein, Oleandrin, *Nerium oleander*, Antiviral, HAART, Apoptosis, HAM/TSP

## Abstract

At present, there are no antiretroviral drugs that inhibit incorporation of the envelope glycoprotein into newly-synthesized virus particles. The botanical glycoside, oleandrin, derived from extracts of Nerium oleander, has previously been shown to reduce the levels of the gp120 envelope glycoprotein on human immunodeficiency virus type-1 (HIV-1) particles and inhibit HIV-1 infectivity *in vitro*. We therefore tested whether oleandrin or an extract from *N. oleander* could also inhibit the infectivity of the human T-cell leukemia virus type-1 (HTLV-1): A related enveloped retrovirus and emerging tropical infectious agent. The treatment of HTLV-1+ lymphoma T-cells with either oleandrin or a *N. oleander* extract did not significantly inhibit viral replication or the release of p19^Gag^-containing particles into the culture supernatants. However, the collected virus particles from treated cells exhibited reduced infectivity on primary human peripheral blood mononuclear cells (huPBMCs). Unlike HIV-1, extracellular HTLV-1 particles are poorly infectious and viral transmission typically occurs via direct intercellular interactions across a virological synapse. We therefore investigated whether oleandrin or a *N. oleander* extract could inhibit virus transmission from a GFP-expressing HTLV-1+ lymphoma T-cell-line to huPBMCs in co-*culture* assays. These results demonstrated that both oleandrin and the crude phytoextract inhibited the formation of virological synapses and the transmission of HTLV-1 *in vitro*. Importantly, these findings suggest oleandrin may have broad antiviral activity against enveloped viruses by reducing the incorporation of the envelope glycoprotein into mature particles, a stage of the infection cycle not targeted by modern HAART.

## INTRODUCTION

The botanical glycoside, oleandrin, and an extract of *Nerium oleander* have been shown to prevent the incorporation of the gp120 envelope glycoprotein of HIV-1 into mature virus particles and inhibit viral infectivity *in vitro* [1]. These findings prompted us to investigate whether oleandrin could similarly inhibit another related retrovirus, the HTLV-1. Growing evidence suggests that certain plant species, including *N. oleander* and *N. indicum*, may contain pharmacological compounds with beneficial medicinal properties [2–12]. Although *N. oleander* is considered poisonous and many of its chemical byproducts elicit cardiotoxicities [13–16], the cardenolides derived from *N. oleander* extracts, including oleandrin, 8-hydroxy-oleandrigenin-3-O- β -Ddiginoside, 5 α -oleaside A, 14-carbonyl-neriaside, 21-hydroxy-neriaside, among others, have been shown to have antiproliferative, anti-inflammatory, and anti-tumorigenic effects [3,4,6,7,17–20]. Indeed, a *N. oleander*-derived drug, PBI-05204, which contains oleandrin as a bioactive agent, has completed both Phase I and initial Phase II clinical trials to test its efficacy and safety in patients with advanced solid cancers [21]. Pan et al. [22] have shown that PBI-05204 inhibited pancreatic tumor growth by targeting the phosphatidylinositol-3-kinase (PI3K)/Akt signaling pathway. Oleandrin has also been shown to accumulate in the central nervous system (CNS) and penetrate the blood-brain-barrier (BBB) following injection [23]; and Garofalo et al. [3] have further demonstrated that oleandrin inhibited tumor growth and disease progression in a murine xenograft model of malignant glioma. Moreover, oleandrin exhibited neuroprotective effects and protected neuronal tissues from damage, due to glucose and oxygen deprivation, dependent on the induction of brain-derived neurotrophic factor (BDNF), in brain slices and *in vivo* models of ischemic stroke [10,24]. It is an intriguing notion that the ability of oleandrin to cross the BBB and inhibit HTLV-1 infectivity and the expression of viral antigens could have potential therapeutic implications for the treatment of HTLV-1-associated neuroinflammatory diseases, including HTLV-1-associated myelopathy/tropical spastic paraparesis (HAM/TSP) [25,26].

The HTLV-1 is a delta oncoretrovirus and blood-borne pathogen that is endemic to tropical equatorial regions, including Southeast Asia (e.g., Japan, China, Taiwan, and Malaysia), Australia and Melanesia, Northern and Central Africa, the Middle East, Central and South America, and certain islands of the Caribbean (e.g., the FWI). It is estimated there are 10–15 million HTLV-1-infected individuals worldwide; and this virus is considered an emerging health threat, as was described in a 2018 open letter to the World Health Organization (WHO) [27]. Indeed, HTLV-1 has been detected with high incidences of seropositivity in remote indigenous populations in central Australia and South America [28,29]. The HTLV-1 infects CD4+ T-lymphocytes and causes adult T-cell leukemia/lymphoma (ATLL)-a rare, yet aggressive hematological malignancy with high rates of therapy-resistance and generally poor clinical outcomes [30–32], in addition to several autoimmune/inflammatory conditions, including infectious dermatitis [33,34], rheumatoid arthritis [35,36], uveitis [37,38], keratoconjunctivitis [39], sicca syndrome [40,41], Sjögren’s syndrome [42,43], and HAM/TSP, among others [25,26,44–46]. The replication of HTLV-1 and the expression of viral antigens is positively and negatively regulated by products encoded by the conserved 3’ pX region, including the viral transactivator protein Tax, the latency-maintenance factors: p30^II^, HBZ, and p13^II^, and the hbz antisense mRNA which can modulate the expression of p30^II^ [47–63]. While ATLL is etiologically linked to viral latency associated with oncogenic transformation and the clonal expansion of HTLV-1-infected cells [30–32], the inflammatory diseases, such as HAM/TSP, are caused by autoimmune and/or immunopathological responses to proviral replication and the expression of viral antigens [25,26,44–46,64–67]. HAM/TSP is a progressive neuroinflammatory disease that results in the deterioration and demyelination of the lower spinal cord. The persistence of proviral replication and the proliferation of HTLV-1-infected cells in the CNS leads to a cytotoxic T-cell response targeted against viral antigens, and which may be responsible for the autoimmune destruction of nervous tissues [25,68–70].

Here we demonstrate that purified oleandrin and a *N. oleander* extract inhibit the infectivity of HTLV-1 particles released into the culture supernatants of treated cells, and also reduce the intercellular transmission of HTLV-1 by inhibiting the Env-dependent formation of virological synapses.

## MATERIALS AND METHODS

### Cell-lines and isolation of primary huPBMCs

The virus-producing HTLV-1-transformed (HTLV-1+) SLB1 lymphoma T-cell-line [71] (kindly provided by P. Green, The Ohio State University-Comprehensive Cancer Center) was cultured in a humidified incubator at 37°C under 10% CO_2_ in Iscove’s Modified Dulbecco’s Medium (IMDM; ATCC No. 30–2005), supplemented with 10% heat-inactivated fetal bovine serum (FBS; Biowest), 100 U/ml penicillin, 100 μg/ml streptomycin-sulfate, and 20 μg/ml gentamycin-sulfate (Life Technologies). Primary human peripheral blood mononuclear cells (huPBMCs) were isolated from whole blood samples, provided without identifiers by the SMU Memorial Health Center under a protocol approved by the SMU Institutional Review Board and consistent with Declaration of Helsinki principles. In brief, 2 ml of whole blood was mixed with an equal volume of sterile phosphate-buffered saline (PBS), pH 7.4, in polypropylene conical tubes (Corning) and then the samples were gently layered over 3 ml of Lymphocyte Separation Medium (MP Biomedicals). The samples were centrifuged for 30 min at 400 × g in a swinging bucket rotor at room temp. The buffy-coat huPBMCs were subsequently aspirated, washed 2X in RPMI-1640 medium (ATCC No. 30–2001), and pelleted by centrifugation for 7 min at 260 × g. The cells were resuspended in RPMI-1640 medium, supplemented with 20% FBS, 100 U/ml penicillin, 100 μg/ml streptomycin-sulfate, 20 μg/ml gentamycin-sulfate, and 50 U/ml recombinant human interleukin-2 (hu-IL-2; Roche Applied Science), and stimulated for 24 h with 10 ng/ml phytohemagglutinin (PHA; Sigma-Aldrich) and grown at 37°C under 10% CO_2_ in a humidified incubator. On the following day, the cells were pelleted by centrifugation for 7 min at 260 × g and washed 2X with RPMI-1640 medium to remove the PHA, and then resuspended and cultured in complete medium, supplemented with antibiotics and 50 U/ml hu-IL-2, as described.

### Generation of GFP-expressing HTLV-1+ SLB1/pLenti-GFP T-cell clones

To generate the GFP-expressing HTLV-1+ SLB1 T-cell clones, 2 × 10^6^ SLB1 cells were plated in 60 mm^2^ tissue-culture dishes (Corning) in IMDM, supplemented with 10% heat-inactivated FBS and antibiotics, and then transduced with lentiviral particles containing a pLenti-6.2/V5-DEST- protein expression vector which also carries a blasticidin-resistance gene [53]. After 6 h, the transduced cells were pelleted by centrifugation for 7 min at 260 × g at room temperature, washed 2X with serum-free IMDM, and resuspended in complete medium supplemented with 5 μg/ml blasticidin (Life Technologies) and aliquoted into 96-well microtiter plates (Corning). The cultures were maintained with blasticidin-selection for two weeks in a humidified incubator at 37°C and 10% CO_2_. The GFP-expressing lymphoblasts were screened by fluorescence-microscopy, and then plated by limiting-dilution in 96-well microtiter plates to obtain homogenous GFP-expressing cell clones. The resulting HTLV-1+ SLB1/pLenti-GFP T-lymphocyte clones were expanded and repeatedly passaged; and the expression of GFP was confirmed by sodium dodecyl sulfate-polyacrylamide gel electrophoresis (SDS-PAGE) and immunoblotting using a rabbit polyclonal Anti-GFP (FL) antibody (Santa Cruz Biotechnology).

### Quantitation of virus production and particle infectivity by Anti-HTLV-1 p19^Gag^ ELISAs

To determine the effects of oleandrin or an extract of *N. oleander* upon HTLV-1 proviral replication and the release of newly-synthesized extracellular virus particles, the HTLV-1+ SLB1 lymphoma T-cell-line was plated at 2 × 10^4^ cells per well in 300 μl of complete medium, supplemented with antibiotics, in 96-well microtiter plates and incubated at 37°C under 10% CO_2_. The purified oleandrin compound and extract of *N. oleander* (Phoenix Biotechnology) [1] were resuspended in the Vehicle solution (20% v/v dimethyl sulfoxide, DMSO, in MilliQ distilled/deionized H_2_O) at a stock concentration of 2 mg/ml and then sterilized using a luer-lock 0.2 μm syringe filter (Millipore). The HTLV-1+ SLB1 cells were treated with oleandrin or the *N. oleander* extract at concentrations of 10, 50, and 100 μg/ml, or with increasing amounts (1.5, 7.5, and 15 μl) of the Vehicle control for 72 h. The 96-well microtiter plates were then centrifuged for 7 min at 260 × g at room temp using an Eppendorf A-2-DWP swinging plate rotor to pellet the cells, and the levels of extracellular p19^Gag^-containing HTLV-1 particles released into the culture supernatants were quantified relative to a p19^Gag^ protein standard by performing colorimetric Anti-p19^Gag^ enzyme-linked immunosorbent assays (ELISAs; Zeptometrix). The samples were analyzed with triplicate replicates on a Berthold Tristar LB 941 multimode microplate-reader at 450 nm in absorbance mode.

To assess the infectivity of newly-synthesized extracellular HTLV-1 particles collected from oleandrin-treated cells, 2 × 10^4^ HTLV-1+ SLB1 T-lymphoblasts were plated in 300 μl of complete medium, supplemented with antibiotics, and the cultures were treated for 72 h with increasing concentrations (10, 50, and 100 μg/ml) of oleandrin or a *N. oleander* extract, or the Vehicle control (1.5, 7.5, and 15 μl). Then, 50 μl of the virus-containing supernatants were used to directly infect huPBMCs plated at a density of 2 × 10^4^ cells per well on 96-well microtiter plates in complete medium, supplemented with antibiotics and hu-IL-2. The oleandrin compound, *N. oleander* extract, or Vehicle control were maintained in the huPBMCs culture medium to control for possible re-infection events by newly-produced particles. After 72 h, the relative levels of extracellular p19^Gag^-containing HTLV-1 virions released into the culture supernatants by the infected huPBMCs were quantified through Anti-HTLV-1 p19^Gag^ ELISAs as described.

### Measuring cellular apoptosis

To assess the relative cytotoxicity of the oleandrin compound, extract of *N. oleander*, or the Vehicle control in treated cell cultures, 2 × 10^4^ HTLV-1+ SLB1 lymphoma T-cells or activated/cultured huPBMCs were plated in 300 μl of complete medium, supplemented with antibiotics, and maintained at 37°C under 10% CO_2_ in a humidified incubator. The cultures were treated with either increasing concentrations (10, 50 and 100 μg/ml) of oleandrin or *N. oleander* extract, or the Vehicle control (1.5, 7.5, 15 μl) and incubated for 72 h. Cyclophosphamide (50 μM; Sigma-Aldrich)-treated cells were included as a positive control for apoptosis. The cells were then aspirated and plated on Permanox 8-chamber tissue-culture slides (Nalge) that had been pre-treated with a sterile 0.01% solution of Poly-L-Lysine and Concanavalin A (1 mg/ml; Sigma-Aldrich). The samples were subsequently stained using a microscopy apoptosis detection kit with Annexin V conjugated to fluorescein isothiocyanate (Annexin V-FITC) and propidium iodide (PI; BD-Pharmingen), and the relative percentages of apoptotic (i.e., Annexin V-FITC and/or PI-positive) cells per field were quantified in-triplicate by confocal fluorescence-microscopy using a 20x objective lens. The total numbers of cells per field were quantified by microscopy using a DIC phase-contrast filter.

### HTLV-1 transmission and virological synapse formation in co-culture assays

As the transmission of HTLV-1 typically occurs through direct contact between an infected cell and uninfected target cell across a virological synapse [72–76], we tested whether oleandrin, a *N. oleander* extract, or the Vehicle control might influence the formation of virological synapses and/or the transmission of infectious HTLV-1 particles via intercellular interactions *in vitro*. For these experiments, 2 × 10^4^ virus-producing HTLV-1+ SLB1 T-cells were plated in 96-well microtiter plates and treated with mitomycin C (100 μg/ml) in 300 μl of complete medium for 2 h at 37°C under 10% CO_2_ [77]. The culture media was then removed, the cells were washed 2X with serum-free IMDM, and the cells were treated for either 15 min or 3 h with increasing amounts (10, 50, and 100 μg/ml) of oleandrin or *N. oleander* extract, or the Vehicle control (1.5, 7.5, and 15 μl). Alternatively, 2 × 10^4^ of the GFP-expressing HTLV-1+ SLB1/pLenti-GFP T-cells were plated on 8-chamber tissue-culture slides in 300 μl of complete medium and treated with mitomycin C, washed 2X with serum-free IMDM, and then treated with oleandrin, *N. oleander* extract, or the Vehicle control as described for confocal microscopy experiments. We next aspirated the medium, washed the HTLV-1+ SLB1 cells 2X with serum-free medium, and added 2 × 10^4^ huPBMCs to each well in 300 μl of RPMI-1640 medium, supplemented with 20% FBS, antibiotics and 50U/ml hu-IL-2, and then co-cultured the cells for another 72 h (the cells were co-cultured for 6 h to visualize virological synapse formation and viral transmission by confocal microscopy using the SLB1/pLenti-GFP lymphoblasts) at 37°C under 10% CO_2_ in a humidified incubator. As a negative control, huPBMCs were cultured alone in the absence of virus-producing cells. The oleandrin, *N. oleander* extract, and Vehicle were maintained in the co-culture medium. The relative levels of extracellular p19^Gag^-containing HTLV-1 particles released into the co-culture supernatants as a result of intercellular viral transmission were quantified by performing Anti-HTLV-1 p19^Gag^ ELISAs. Virological synapses formed between the GFP-positive HTLV-1+ SLB/pLenti-GFP cells and huPBMCs were visualized using immunofluorescence-confocal microscopy by staining the fixed samples with an Anti-HTLV-1 gp21^Env^ primary antibody and a rhodamine red-conjugated secondary antibody. Diamidino-2-phenyl-indole, dihydrochloride (DAPI; Molecular Probes) nuclear-staining was included for comparison and to visualize uninfected (i.e., HTLV-1-negative) cells. The intercellular transmission of HTLV-1 to the huPBMCs in co-culture assays was quantified by counting the relative percentages of HTLV-1 gp21^Env^-positive (and GFP-negative) huPBMCs in 20 visual fields using a 20x objective lens.

### Microscopy

The Annexin V-FITC/PI-stained samples to quantify cellular apoptosis and cytotoxicity were visualized by confocal fluorescence-microscopy on a Zeiss LSM800 instrument equipped with an Airyscan detector and stage CO_2_ incubator, using a Plan-Apochromat 20 × 0.8 objective lens and Zeiss ZEN system software (Carl Zeiss Microscopy). The formation of virological synapses and viral transmission (i.e., determined by quantifying the relative percentages of Anti-HTLV-1 gp21^Env^-positive huPBMCs) between the mitomycin C-treated HTLV-1+ SLB1/pLenti-GFP lymphoblasts and cultured huPBMCs were visualized by immunofluorescence-confocal microscopy using a Plan-Apochromat 20 × 0.8 objective lens. The relative fluorescence-intensities of the DAPI, Anti-HTLV-1 gp21^Env^-specific (rhodamine red-positive), and GFP signals were graphically quantified using the Zen 2.5D analysis tool (Carl Zeiss Microscopy). The GFP-expressing HTLV-1+ SLB1/pLenti-GFP T-cell clones were screened by confocal fluorescence-microscopy on a Nikon Eclipse TE2000-U inverted microscope and D-Eclipse confocal imaging system, equipped with 633 nm and 543 nm He/Ne and 488 nm Ar lasers, using a Plan Fluor 10 × 0.30 objective lens and DIC phase-contrast filter (Nikon Instruments).

### Data analysis

The statistical significance of experimental data sets was determined using unpaired two-tailed Student’s t-tests (alpha =0.05) and calculated p values using the Shapiro-Wilk normality test and Graphpad Prism 7.03 software. The p values were defined as: 0.1234 (ns), 0.0332 (*), 0.0021 (**), 0.0002 (***), <0.0001 (****). Unless otherwise noted, error bars represent the SEM from at least three independent experiments.

## RESULTS

### The botanical glycoside oleandrin does not inhibit viral replication or the release of p19Gag-containing HTLV-1 particles

Oleandrin is a lipid-soluble, botanical cardiac glycoside comprised of: (1) the steroid aglycone, oleandrigenin, and (2) a sugar moiety (e.g., D-diginosyl) and its chemical structure is shown in Figure 1a [1,4,78]. To determine whether the purified oleandrin compound, or an extract of *N. oleander*, could inhibit HTLV-1 proviral replication and/or the production and release of p19^Gag^-containing virus particles, the virus-producing HTLV-1-transformed SLB1 lymphoma T-cell-line [71] was treated with increasing concentrations of oleandrin or a *N. oleander* extract, or the sterile Vehicle control (20% DMSO in MilliQ-treated ddH_2_O) and then incubated for 72 h at 37°C under 10% CO_2_. The cells were later pelleted by centrifugation and the relative levels of extracellular p19^Gag^-containing virus particles released into the culture supernatants were quantified by performing Anti-HTLV-1 p19^Gag^ ELISAs (Zeptometrix). The results in Figure 1b demonstrate that neither oleandrin, nor the *N. oleander* extract, significantly inhibited viral replication or the production and release of p19^Gag^-containing virions relative to untreated cells or the Vehicle negative control (note: the data are presented on an abbreviated scale and the concentrations of HTLV-1 p19^Gag^ in the culture supernatants were determined relative to a p19^Gag^ protein standard).

We next assessed the cytotoxicity of the different dilutions of the purified oleandrin compound and *N. oleander* extract in treated HTLV-1+ SLB1 lymphoblast cultures. For these experiments, SLB1 T-cells were treated with increasing concentrations (10, 50, and 100 μg/ml) of oleandrin or a *N. oleander* extract for 72 h as described above. As a negative control, the cells were also treated with increasing amounts (1.5, 7.5, and 15 μl) of the Vehicle solution which corresponded to the volumes used in the drug-treated cultures. Cyclophosphamide-treated SLB1 cells were included as a positive control for apoptosis. Then, the samples were washed and stained with Annexin V-FITC and propidium iodide (PI) and analyzed by confocal fluorescence-microscopy. The relative percentages of apoptotic (i.e., Annexin V-FITC and/or PI-positive) cells per field were quantified in-triplicate using a 20x objective lens, as compared to the total numbers of cells visualized using a DIC phase-contrast filter. These results (Figures 1c and 1d) revealed that the lowest concentration (10 μg/ml) of oleandrin and the *N. oleander* extract did not induce significant cytotoxicity/apoptosis. However, the higher concentrations (50 and 100 μg/ml) of the crude phytoextract induced notably more apoptosis than did the oleandrin compound (Figures 1c and 1d). This is consistent with the fact that oleandrin represents only 1.23% of the *N. oleander* extract [1] which also contains other toxic cardenolides, including folinerin and digitoxigenin [13]. The cytotoxicity caused by oleandrin was not significantly higher than the Vehicle control in treated HTLV-1+ SLB1 cells (Figures 1c and 1d).

### Oleandrin and the *N. oleander* extract inhibit the infectivity of p19^Gag^-containing HTLV-1 particles released into the culture supernatants of treated cells

Singh et al. [1] have previously demonstrated that oleandrin and a *N. oleander* extract inhibited the incorporation of the gp120 envelope glycoprotein into newly-synthesized HIV-1 particles and reduced the infectivity of virions released from treated cells. We therefore tested whether these agents could inhibit the infectivity of newly-synthesized extracellular p19^Gag^-containing HTLV-1 particles obtained from treated SLB1 T-cells. For these studies, HTLV-1+ SLB1 lymphoma T-cells were treated with increasing concentrations of either the oleandrin compound or *N. oleander* extract, or the Vehicle control for 72 h in 96-well microtiter plates, and then the virus-containing supernatants were collected and used to directly infect primary cultured, human peripheral blood mononuclear cells (huPBMCs) *in vitro*. Following 72 h, the relative levels of extracellular p19^Gag^-containing virus particles released into the culture supernatants, as a result of direct infection, were quantified by performing Anti-HTLV-1 p19^Gag^ ELISAs. Interestingly, the lowest concentration (10 μg/ml) of both oleandrin and the *N. oleander* extract inhibited the infectivity of newly-synthesized p19^Gag^-containing virus particles released into the culture supernatants of treated cells, relative to a comparable amount of the Vehicle control (Figure 2a). The levels of inhibition, however, seemed to plateau and did not continue to increase with higher concentrations, which suggests their molecular target(s) could be limiting and/or saturated at the concentrations tested (Figure 2a).

We also assayed the cytotoxicity of purified oleandrin and the *N. oleander* extract, compared to the Vehicle negative control, in treated huPBMCs. Primary buffy-coat huPBMCs were isolated and stimulated with phytohemagglutinin (PHA) and cultured in the presence of recombinant human interleukin-2 (hIL-2). The cells were then treated for 72 h with increasing concentrations of oleandrin or a *N. oleander* extract, or with increasing volumes of the Vehicle. The samples were subsequently stained with Annexin V-FITC and PI and the relative percentages of apoptotic (i.e., Annexin V-FITC and/or PI-positive) cells per field were quantified by confocal fluorescence-microscopy and counting in-triplicate. As shown in Figure 2b, the oleandrin compound exhibited moderate cytotoxicity (e.g., 35–37% at the lowest concentration) in huPBMCs as compared to the Vehicle control. By contrast, the *N. oleander* extract was significantly cytotoxic and induced high levels of programmed cell-death even at the lowest concentration (Figure 2b). The huPBMCs were somewhat more sensitive to purified oleandrin than the HTLV-1+ SLB1 lymphoblasts; however, the huPBMCs were drastically more sensitive to the crude *N. oleander* extract which also contains other cytotoxic compounds [13].

### Oleandrin inhibits Env-dependent virological synapse formation and the transmission of HTLV-1 in co-culture assays

Unlike HIV-1, HTLV-1 particles are poorly infectious and viral transmission typically occurs via intercellular interactions across a virological synapse [72–76]. Therefore, we next investigated whether oleandrin or the *N. oleander* extract could interfere with the transmission of HTLV-1 particles to target huPBMCs in co-culture experiments. For these studies, the virus-producing HTLV-1+ SLB1 T-cell-line was treated with mitomycin C [77] and then with increasing amounts of oleandrin, *N. oleander* extract, or the Vehicle control for either 15 min or 3 h. The SLB1 cells were washed 2X with serum-free medium and equivalent numbers of huPBMCs were then added to each well, and the samples were co-cultured for 72 h in complete medium at 37°C under 10% CO_2_ in a humidified incubator. The relative intercellular transmission of HTLV-1 was assessed by performing Anti-HTLV-1 p19^Gag^ ELISAs to measure the levels of extracellular virus released into the culture supernatants. Interestingly, the results in Figure 3a demonstrate that both oleandrin and the *N. oleander* extract inhibited the transmission of HTLV-1 as compared to the Vehicle control –although there were no differences observed between the 15 min and 3 h of pre-treatment of the HTLV-1+ SLB1 cells.

To directly visualize the formation of virological synapses and the interaction of target huPBMCs with the virus-producing cells, we generated HTLV-1+ SLB1 T-cell clones that stably express the Green fluorescent protein (GFP; Figure 3b). The HTLV-1+ SLB1 lymphoma T-cell-line was transduced with lentiviral-GFP particles and the GFP-positive cell clones were selected on blasticidin, and subsequently confirmed by direct fluorescence-microscopy and immunoblotting (Figure 3b). The formation of virological synapses between the huPBMCs and mitomycin C-treated HTLV-1+ GFP-expressing SLB1 cells was visualized by staining the samples with an Anti-HTLV-1 gp21^Env^ primary antibody and rhodamine-red-conjugated secondary antibody, together with DAPI nuclear-staining, and then performing confocal immunofluorescence-microscopy. The arrows in the top panels of Figure 3c indicate virological synapses between the huPBMCs (i.e., gp21^Env^-positive; GFP-negative) and HTLV-1+ SLB1 cells treated with the Vehicle control. There were markedly fewer synapses observed in the samples treated with oleandrin or the *N. oleander* extract (Figures 3c). This was further evidenced by the reduced percentages of HTLV-1 gp21^Env^-positive huPBMCs in these treated samples as compared to the Vehicle control (Figure 3c).

## DISCUSSION

Oleandrin and other structurally similar digoxin-related cardenolides exert most of their toxic effects by inhibiting sodium/potassium pump ATPase catalytic activity [5–7,79]. Conversely, Yang et al. [80] have shown that the inhibition of sodium/potassium pump ATPase activity by oleandrin reduced the sensitivity of swine testicular cells to infection by the porcine transmissible gastroenteritis virus *in vitro*. Oleandrin has also been demonstrated to inhibit nuclear factor-kappa B (NF-B) and activator protein-1 (AP-1)-dependent transcriptional signaling through the c-Jun NH2-terminal kinase (JNK) and mitogen-activated/extracellular signal-regulated kinase (MEK) pathways, and thus could have anti-inflammatory and anti-tumorigenic properties [81,82]. Indeed, Afaq et al. [19] have shown that topically-applied oleandrin inhibited 12-O-tetradecanoylphorbol-13-acetate (TPA)-induced inflammation and tumor promotion in a murine model of skin cancer. While the mechanism(s) by which oleandrin interferes with NF-КB-mediated inflammatory signaling remains to be fully determined, Manna et al. [83] have suggested oleandrin could inhibit interleukin-8 (IL-8)-receptor functions by altering plasma membrane fluidics and downregulating this cytokine receptor from the cell surface. Oleandrin also induces the production and accumulation of damaging reactive oxygen species (ROS) which could contribute to its overall cytoxicity and anti-tumorigenic effects [20]. Turan et al. [8] have also demonstrated that extracts of *N. oleander* resulted in decreased levels of the P-glycoprotein on the surfaces of treated leukemic cell-lines. Though speculative, it is likely that oleandrin’s ability to inhibit the incorporation of envelope glycoproteins into budding virions could reflect oleandrin-induced alterations to the plasma membrane and vesicle fluidics in a manner analogous to the downregulation of P-glycoprotein and the IL-8-receptor [8,83].

It is possible the inhibition of HTLV-1-infectivity, viral transmission and virological synapse formation by oleandrin could have implications for the suppression of viral antigen expression in HTLV-1-infected patients with HAM/TSP [25,26]. There are currently no good therapeutic options for the treatment of HTLV-1-associated neuroinflammatory diseases caused by the infiltration of HTLV-1-infected CD4+ lymphocytes into the CNS [64–66]. HTLV-1-associated myelopathy/tropical spastic paraparesis is a progressive neurodegenerative disease that leads to the deterioration and demyelination/atrophy of the lower spinal cord as a result of inflammatory immunopathological responses to viral antigens [68,70,84–87]. Anderson et al. [68] have demonstrated that viral antigens, including the transactivator protein Tax, were present in exosomes in the cerebral spinal fluid (CSF) of HAM/TSP patients. Also, Fox et al. [88] have detected extrachromosomal long terminal repeat (LTR) circular DNA indicative of virus replication in PBMCs isolated from HTLV-1-infected HAM/TSP patients. Oleandrin has been shown to penetrate the blood-brain-barrier and accumulate in brain tissues following injection [23], suggesting this compound may be able to target HTLV-1-infected lymphocytes in the CNS to potentially help combat HAM/TSP. Moreover, Dunn et al. [24] have shown that oleandrin is the critical neuroprotective component of the botanical drug PBI-05205, and that oleandrin prevented neuronal apoptosis due to oxygen and glucose deprivation in brain slice and animal models of ischemic injury. This neuroprotective effect was attributed to the induction of BDNF by oleandrin [10]. The induction of BDNF was also shown to be central for the anti-tumorigenic activity of oleandrin in a murine model of glioma [2,3].

These findings, taken together with the observations of Singh et al. [1], suggest the botanical glycoside oleandrin may have broad antiretroviral activity by reducing the incorporation of the envelope glycoprotein into newly-synthesized virus particles –a stage of the infection cycle that is not targeted by modern HAART. Jejcic et al. [89] have previously demonstrated that a synthetic peptide mimetic, glycyl-prolyl-glycine amide (GPG-NH(2)), prevented the maturation of the HIV-1 envelope glycoprotein into gp120/gp41 and caused it to become degraded in the endoplasmic reticulum, and resulted in the production of defective virus particles with reduced envelope-incorporation and decreased infectivity. Intriguingly, Rajbhandari et al. [11] have shown that extracts from a related botanical, Nerium indicum, possessed antiviral activity against influenza virus (A/WSN/33 (H1N1) London isolate) and herpes simplex virus type-1 (HSV-1, KOS strain) *in vitro*. James et al. [90] have further reported that oleandrin, as well as an extract of N. oleander, inhibited virus production in treated Vero cells infected with either the Marburg or Ebola filoviruses, or Venezuelan equine encephalitis virus.

## CONCLUSION

It is therefore possible that phytomedicinal compounds, such as the cardenolide oleandrin, may one day represent a cost-effective therapeutic strategy to help combat enveloped virus infections in developing countries with limited access to modern antivirals.

## Figures and Tables

**Figure 1a:**
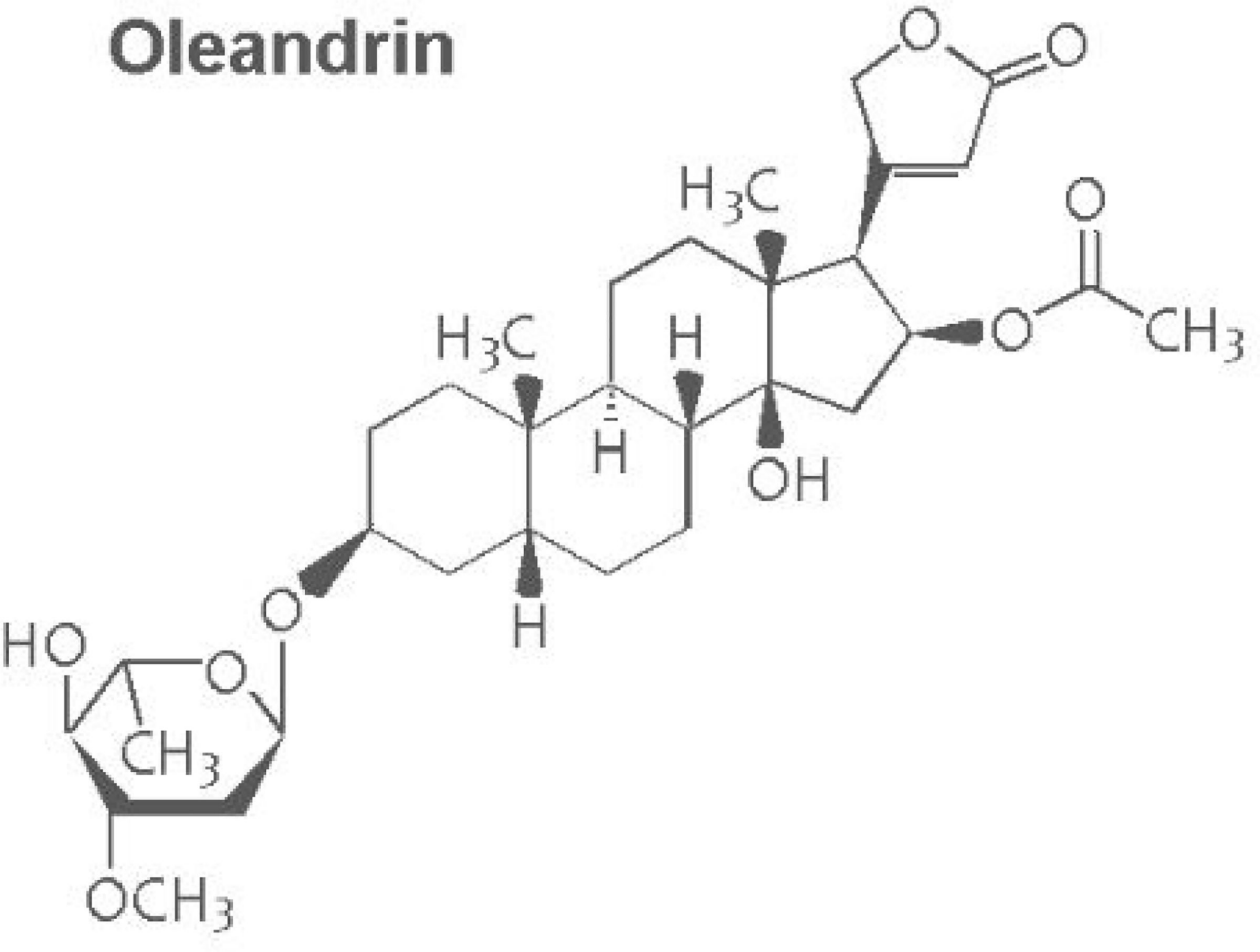
Diagram of the chemical structure of oleandrin.

**Figure 1b:**
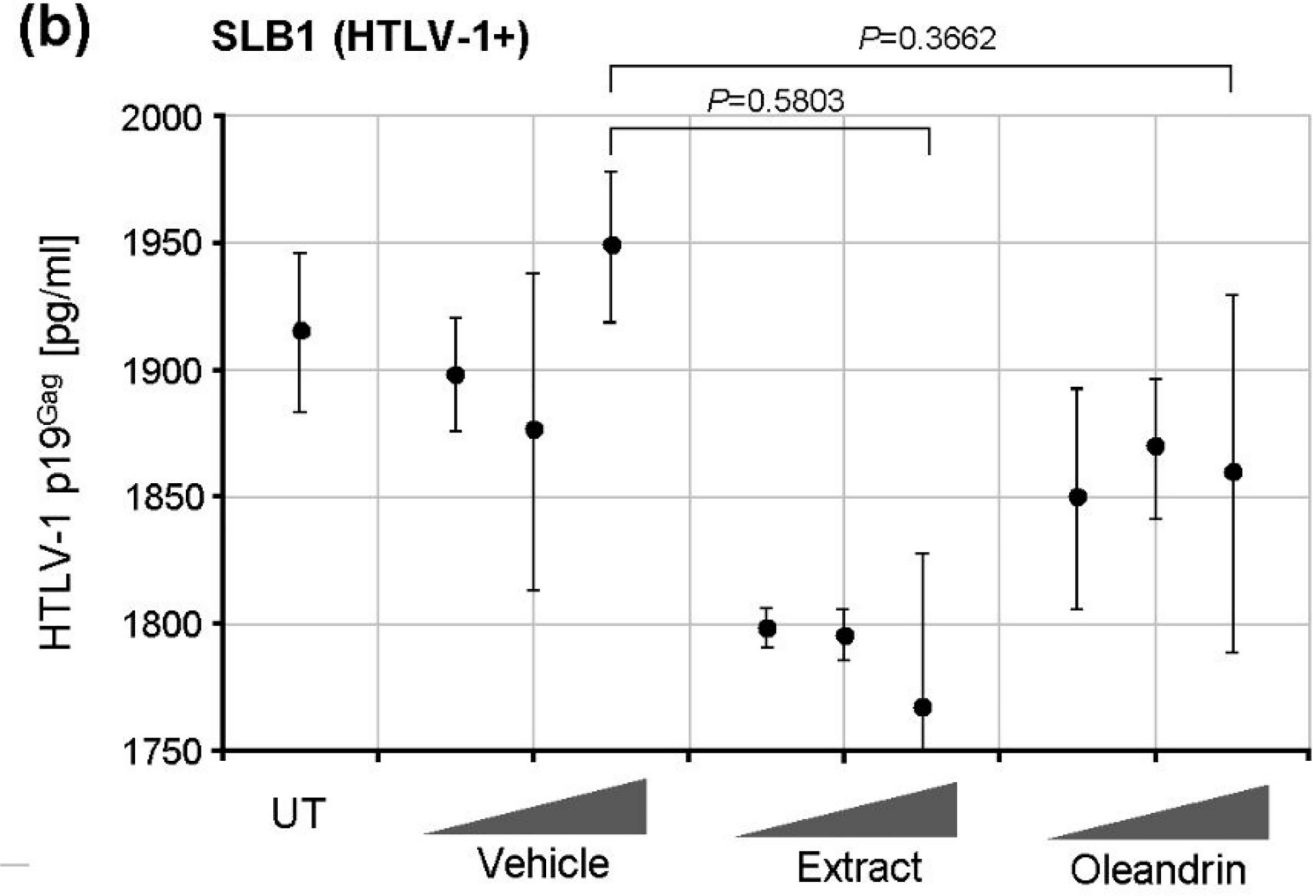
The HTLV-1+ SLB1 lymphoma T-cell-line was treated for 72 h with the Vehicle control (1.5 ml, 7.5 ml, or 15 ml), or increasing concentrations (10 mg/ml, 50 mg/ml, and 100 mg/ml) of the oleandrin compound or an extract of N. oleander. Viral replication and the release of extracellular particles into the culture supernatants were quantified by performing Anti-HTLV-1 p19Gag ELISAs (Zeptometrix). Untreated (UT) cells are shown for comparison.

**Figure 1c:**
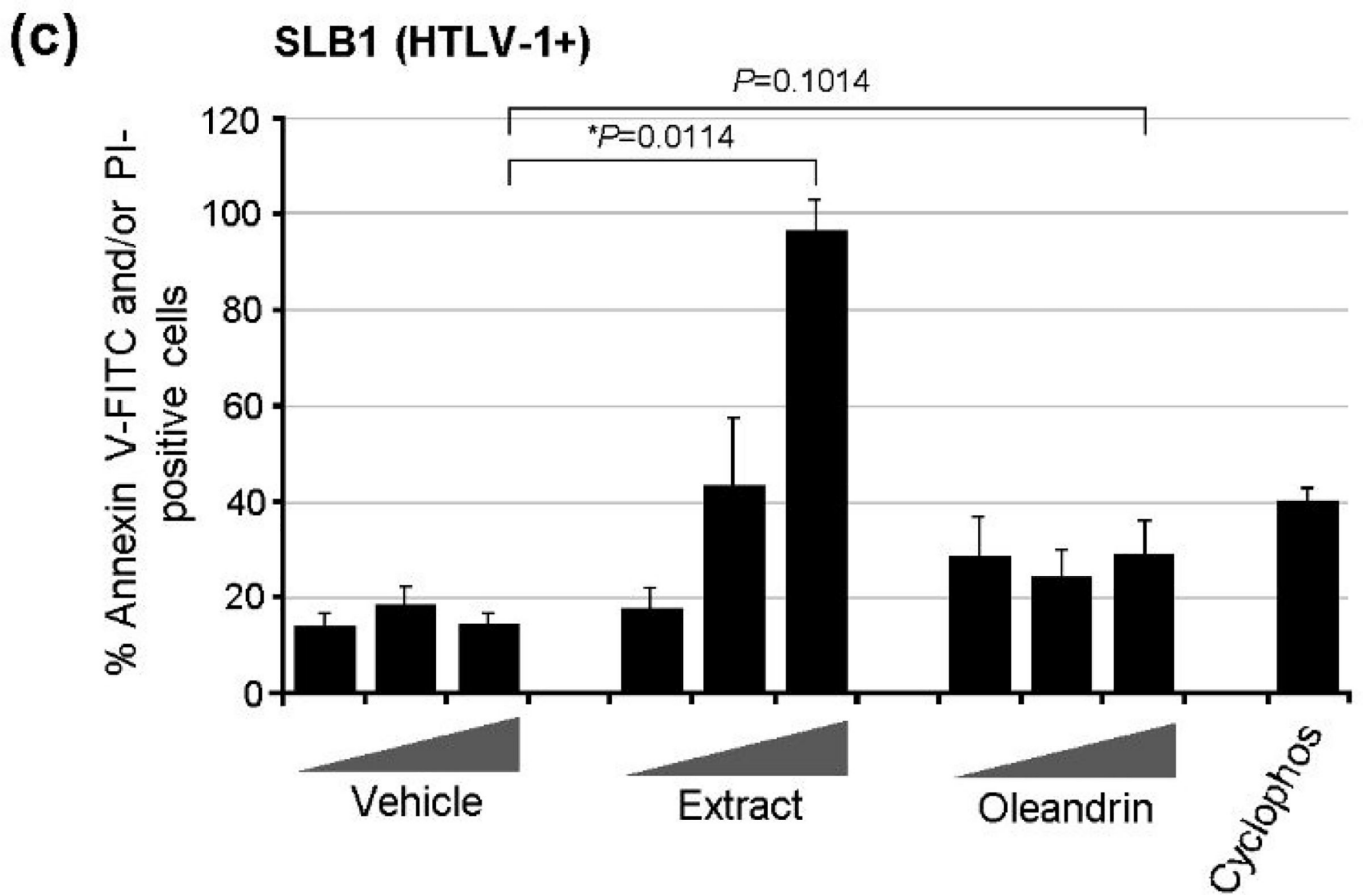
The cytotoxicity of the Vehicle control, oleandrin, and N. oleander extract was measured by treating the HTLV-1+ SLB1 lymphoma T-cell-line as in a and then staining the cultures with Annexin V-FITC and propidium iodide (PI). Cyclophosphamide (50 mM; Sigma-Aldrich)-treated cells were included as a positive control for apoptosis. The relative percentages of Annexin V-FITC and/or PI-positive cells were quantified by fluorescence-microscopy and counting triplicate visual fields using a 20x objective lens.

**Figure 1d:**
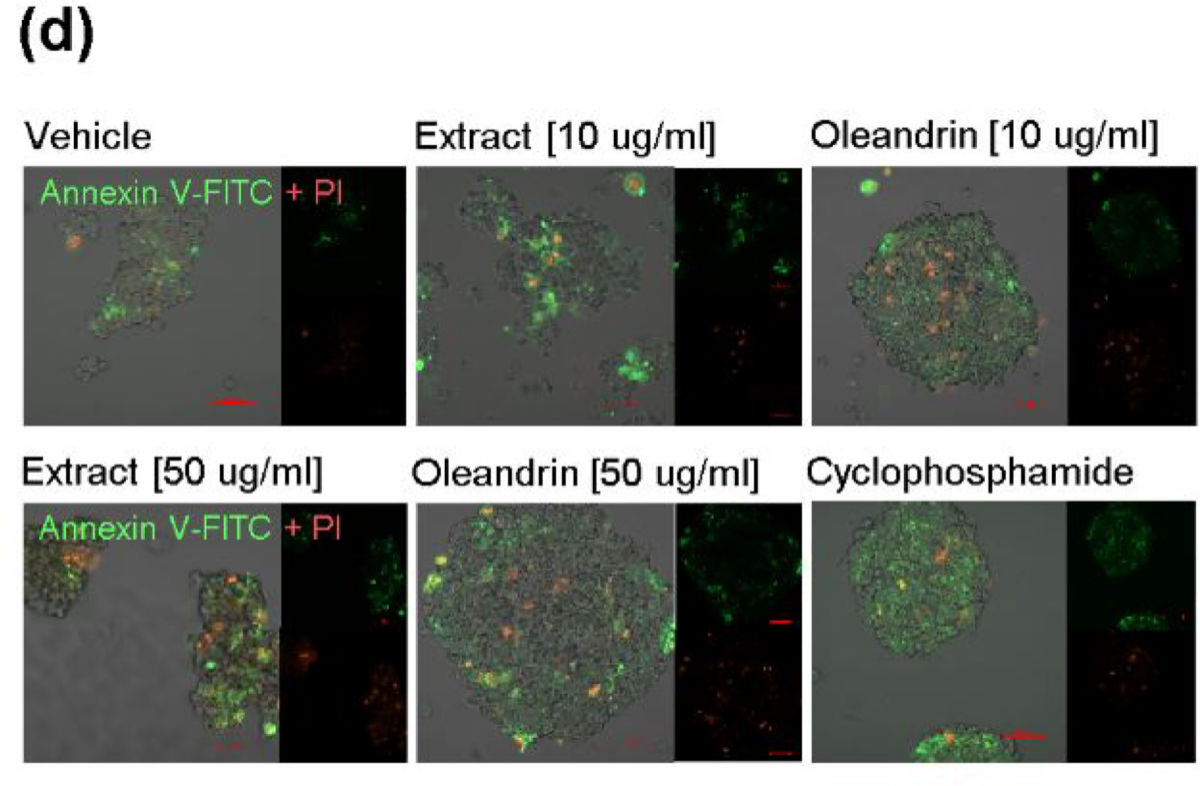
Representative micrographs of the Annexin V-FITC (green) and PI (red)-staining results with DIC phase-contrast in the merged images are shown. The individual Annexin V-FITC and PI fluorescent channel images are also provided. Scale bar, 20 mm. All the data is representative of at least three independent experiments, and the data in b and c represent the mean of the experiments ± standard deviation (error bars).

**Figure 2a:**
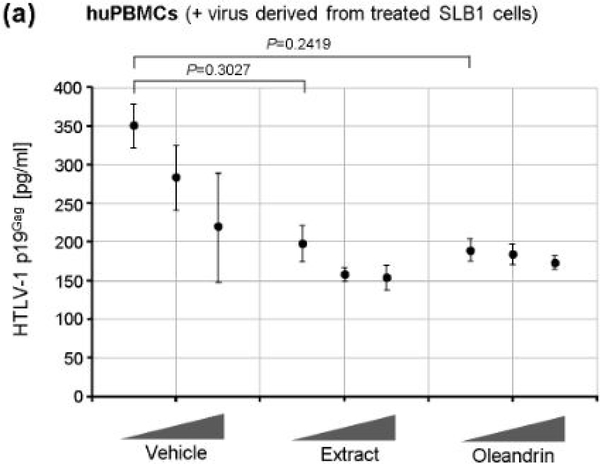
The HTLV-1+ SLB1 lymphoma T-cell-line was treated with the Vehicle control, or increasing concentrations (10 mg/ml, 50 mg/ml, and 100 mg/ml) of the N. oleander extract or oleandrin compound for 72 h and then the virus-containing supernatants were collected and used to directly infect primary huPBMCs. The Vehicle control, N. oleander extract, or oleandrin were also included in the culture media for the huPBMCs. After 72 h, the culture supernatants were collected and the relative amounts of extracellular virus particles produced were quantified by performing Anti-HTLV-1 p19Gag ELISAs.

**Figure 2b:**
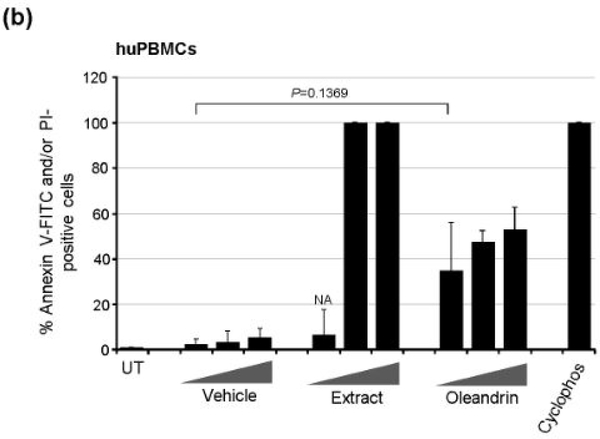
The cytotoxic effects of the Vehicle control, N. oleander extract, and the oleandrin compound were assessed by treating primary huPBMCs for 72 h as in a, and then the cultures were stained with Annexin V-FITC and PI. The relative percentages of apoptotic (i.e., Annexin V-FITC and/or PI-positive) cells were quantified by fluorescence-microscopy and counting triplicate visual fields using a 20x objective lens. The total numbers of cells were determined using DIC phase-contrast microscopy. Cyclophosphamide (50 mM)-treated cells were included as a positive control for apoptosis. NA, the number of cells in this sample was too low for accurate assessment due to higher toxicity. The data in a and b represent the mean ± standard deviation (error bars) from three independent experiments.

**Figure 3a:**
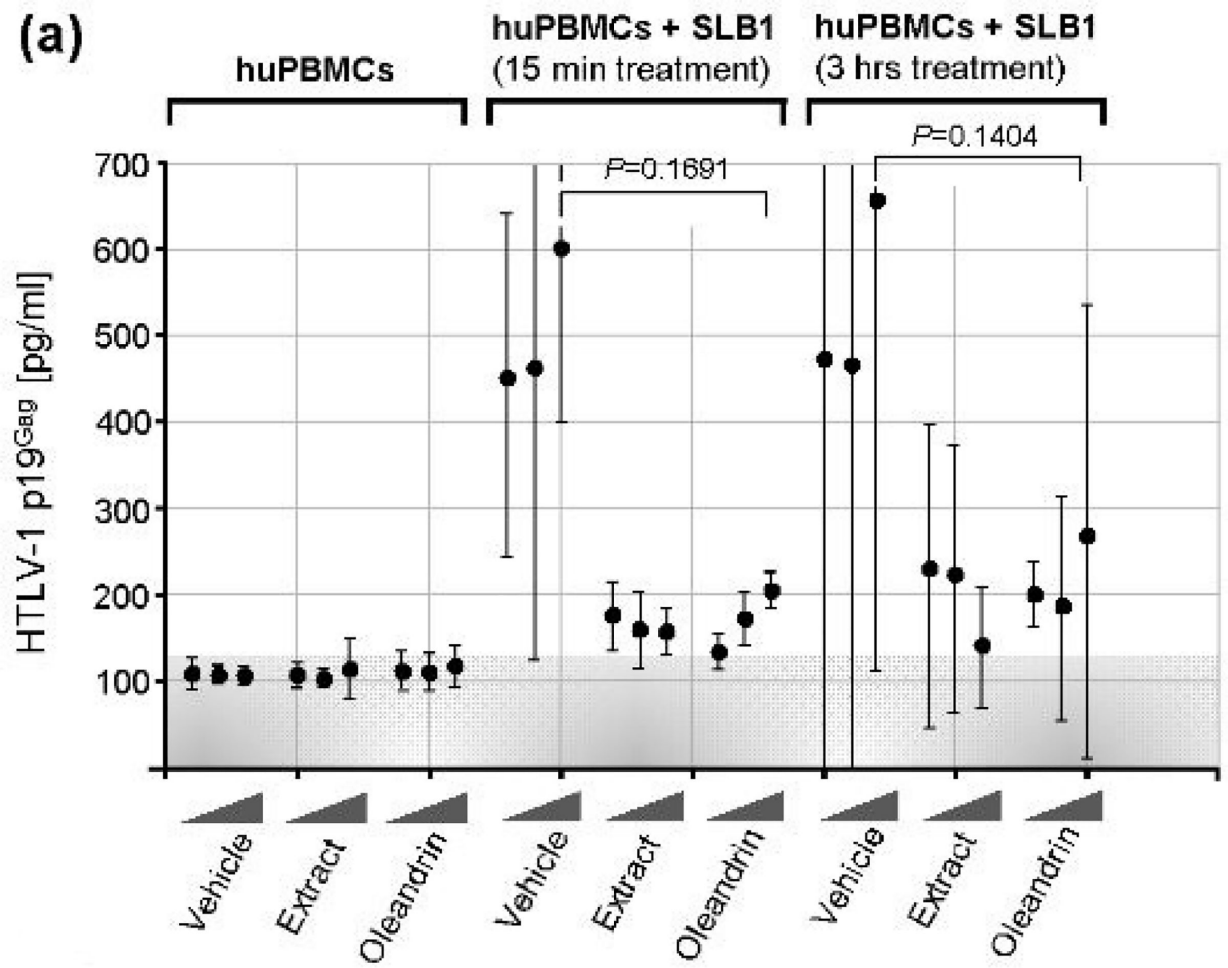
Primary huPBMCs were co-cultured with mitomycin C-treated HTLV-1+ SLB1 lymphoma T-cells which were pre-treated for either 15 min or 3 h with the Vehicle control, or increasing concentrations (10 mg/ml, 50 mg/ml, and 100 mg/ml) of the N. oleander extract or oleandrin compound. The Vehicle control, extract, and compound were also present in the co-culture media. After 72 h, the supernatants were collected and the amounts of extracellular virus particles released were quantified by performing Anti-HTLV-1 p19Gag ELISAs. The data represent the mean ± standard deviation (error bars) from three independent experiments.

**Figure 3b:**
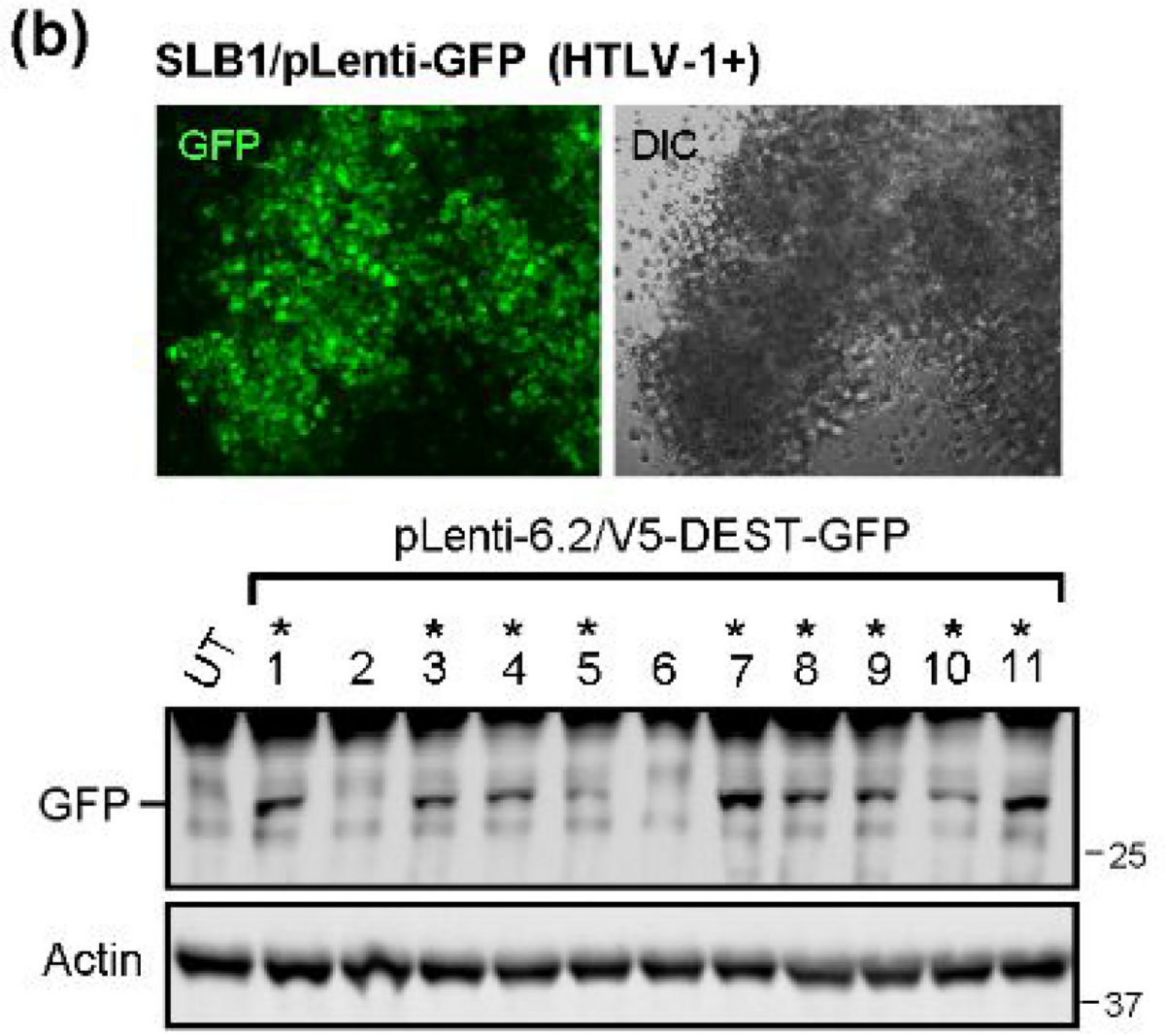
A GFP-expressing HTLV-1+ SLB1 T-cell-line was generated by transducing SLB1 lymphoma T-cells with a pLenti-6.2/V5-DEST-GFP vector with selection on blasticidin (5 mg/ml; Life Technologies) for two weeks. The GFP-positive clones were screened by fluorescence-microscopy (top panels) and immunoblotting (lower panels), and expanded and repeatedly passaged. The DIC phase-contrast image is provided for comparison.

**Figure 3c:**
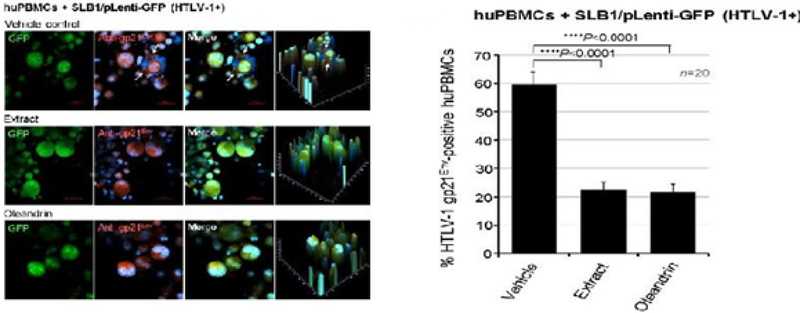
The formation of virological synapses between huPBMCs and the mitomycin C-treated HTLV-1+ SLB1/pLenti-GFP lymphoblasts (green cells) that had been pre-treated for 3 h with the Vehicle control or increasing amounts (10 μg/ml, 50 μg/ml, and 100 μg/ml) of the N. oleander extract or oleandrin compound were visualized by fluorescence-microscopy. Virus transmission was assessed by quantifying the relative percentages of infected (i.e., HTLV-1 gp21-positive, red) huPBMCs (GFP-negative) in 20 visual fields (n=20) by fluorescence-microscopy using a 20x objective lens (see arrows in the Vehicle control panels in c). The averaged data with standard deviation (error bars) are shown in d.
